# Supplementary material for: Conditional chemoconnectomics (cCCTomics) as a strategy for efficient and conditional targeting of chemical transmission
Source: eLife. 2024 Apr 30;12:RP91927. doi: 10.7554/eLife.91927 (PMC11060718; doi:10.7554/eLife.91927)
Supplement: Figure 3—source data 3. [file elife-91927-fig3-data3.zip › Figure 3 source data3 part 1.zip!/Figure 3-Source data 3-part1/1063-13-CRISPResso_Report_8cz_fsdr.zip!/CRISPResso_on_1063_14/2b.Nucleotide_percentage_quilt_around_sgRNA_cgtcgagcacatgacatcaa.pdf]

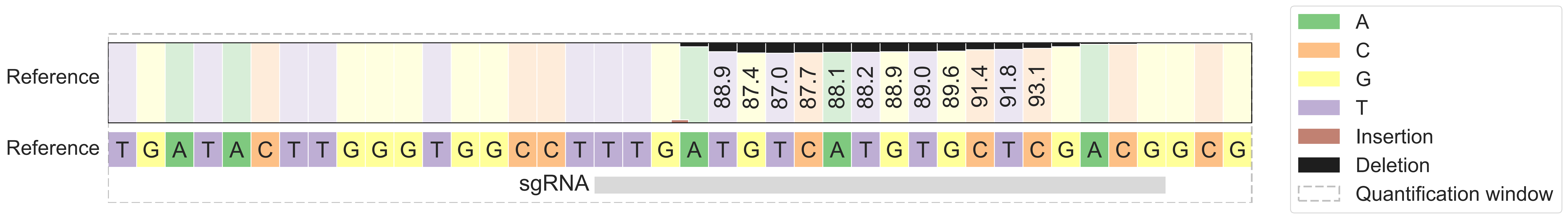

Reference
Reference
T G A T A C T T G G G T G G C C T T T G A T G T C A T G T G C T C G A C G G C G
88.9 87.4 87.0 87.7 88.1 88.2 88.9 89.0 89.6 91.4 91.8 93.1
sgRNA
A
C
G
T
Insertion
Deletion
Quantification window
